# Supplementary material for: Mutation in the Ciliary Protein C2CD3 Reveals Organ-Specific Mechanisms of Hedgehog Signal Transduction in Avian Embryos
Source: J Dev Biol. 2021 Mar 25;9(2):12. doi: 10.3390/jdb9020012 (PMC8103285; doi:10.3390/jdb9020012)
Supplement: Supplementary file 1 [file jdb-09-00012-s001.pdf]

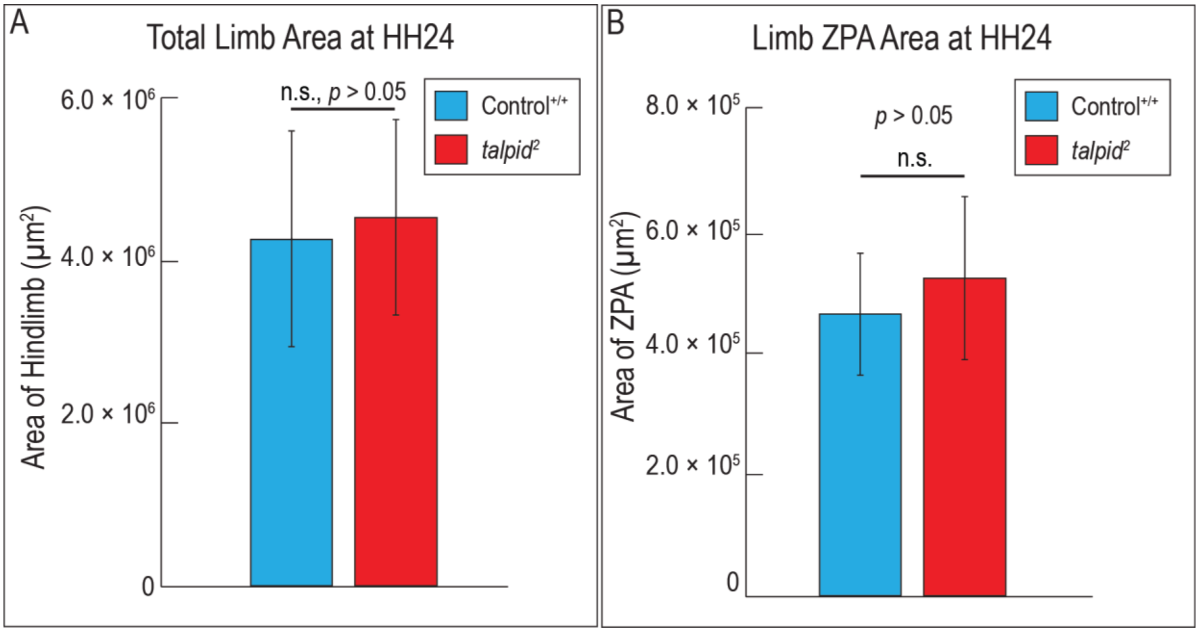

Supplementary Figure S1. Size of hindlimbs and ZPA are not significantly different between control$^{+/+}$ and *ta*^{2} embryos.(A) Quantification of hindlimb area in HH24 control$^{+/+}$ (n = 6) and *ta*^{2} (n = 6) limbs. (B) Quantification of ZPA area in HH24 control$^{+/+}$ (n = 9) and *ta*^{2} (n = 9) limbs. Error bars represent the mean data ± s.d. Statistical analysis was performed utilizing Student's *t*-test (n.s. denotes no significance at .05 significance level).

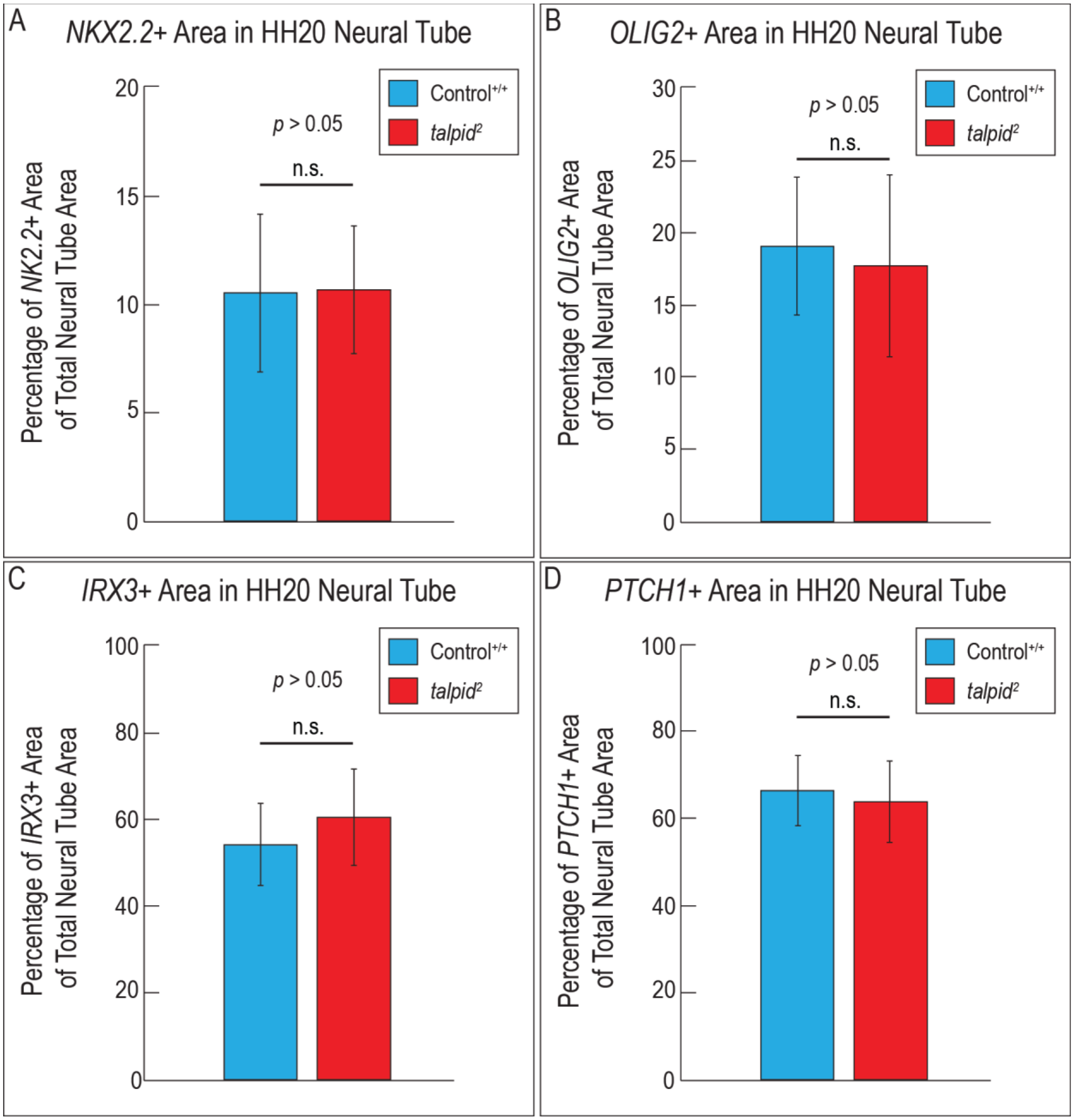

Supplementary Figure S2. No significant difference in the ratios of expression of neural progenitors and *PTCH1* between control$^{+/+}$ and *ta*^{2} neural tubes. (A-D) Quantification of (A) *NKX2.2*+ area, (B) *OLIG2*+ area, (C) *IRX3*+ area, and (D) *PTCH1*+ area in HH20 control$^{+/+}$ and *ta*^{2} neural tubes expressed as a percentage of total neural tube area. Error bars represent the mean data ± s.d. Statistical analysis was performed utilizing Student's *t*-test (n.s. denotes no significance at .05 significance level).

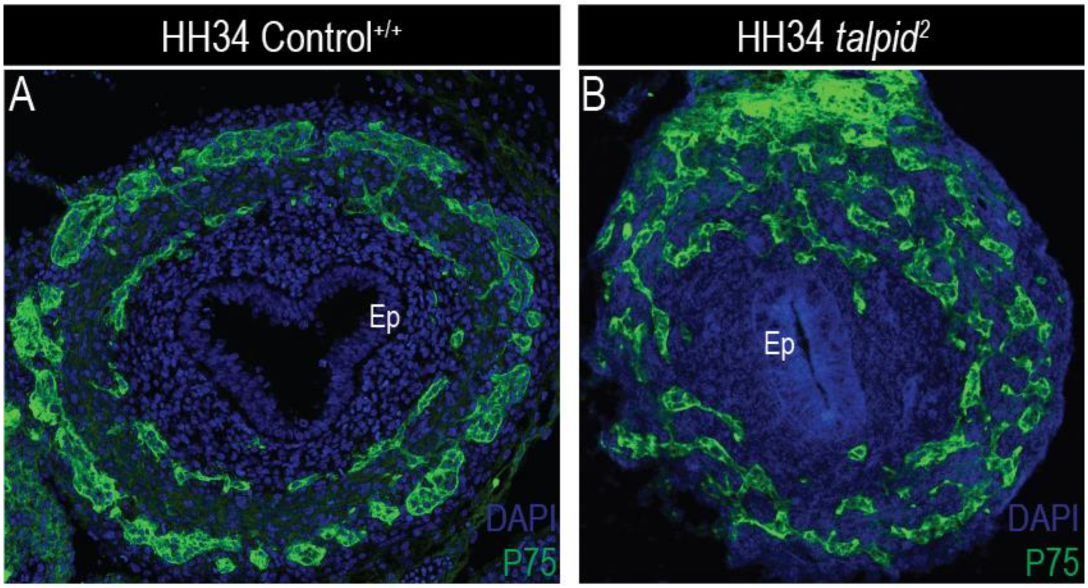

Supplementary Figure S3. ENCC migration is perturbed in the *ta*^{2} midgut. (A-B) Immunostaining for P75 in HH34 (A) control$^{+/+}$ and (B) *ta*^{2} midguts. Ep: intestinal epithelium.

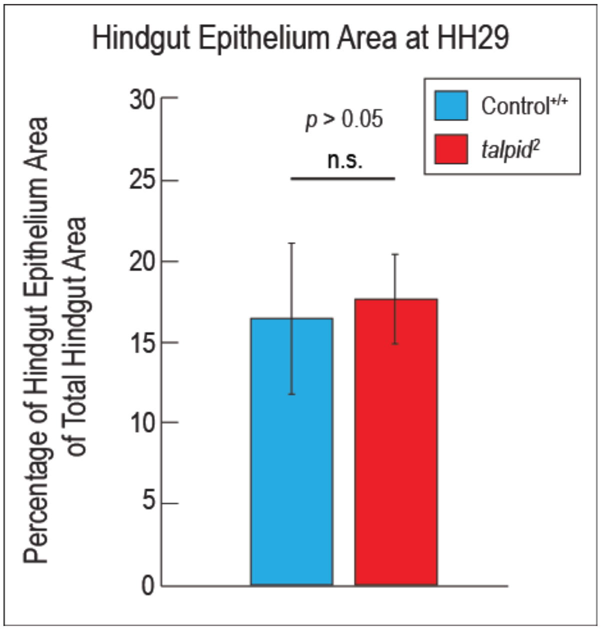

Supplementary Figure S4. The area of the *SHH*+ epithelium in the *ta*^{2} hindgut is not significantly different from control$^{+/+}$ embryos. Quantification of the hindgut epithelium area in HH29 control$^{+/+}$ and *ta*^{2} hindguts expressed as a percentage of total hindgut area. Error bars represent the mean data ± s.d. Statistical analysis was performed utilizing Student's *t*-test (n.s. denotes no significance at .05 significance level).

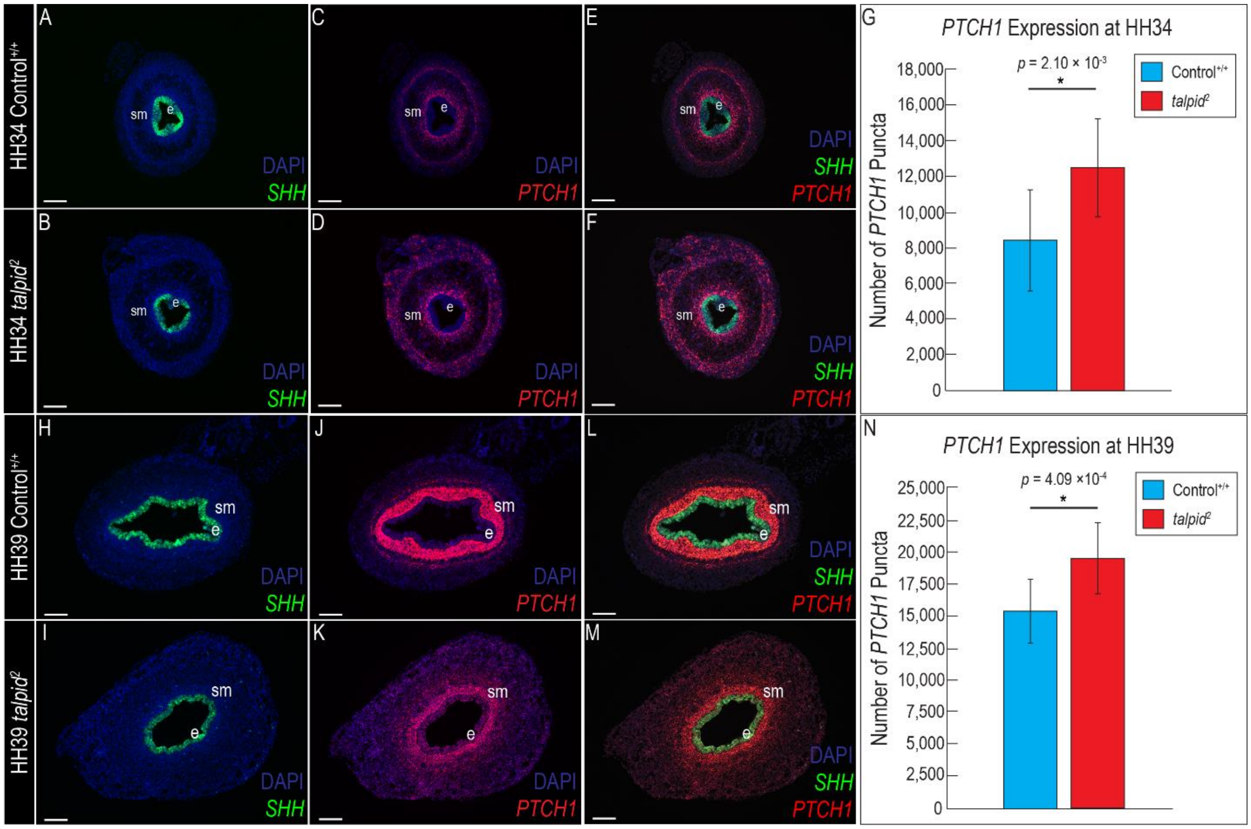

Supplementary Figure S5. Hh signaling activity in the *ta*^{2} hindgut is increased in several developmental stages. (A-F) RNAscope *in situ* hybridization for (A, B) *SHH*, (C, D), *PTCH1* or (E, F) both *SHH* and *PTCH1* in HH34 control$^{+/+}$ and *ta*^{2} hindguts. (G) Counts for *PTCH1* puncta in HH34 control$^{+/+}$ and *ta*^{2} hindguts. (H-M) RNAscope *in situ* hybridization for (H, I) *SHH*, (J, K) *PTCH1* or (L, M) both *SHH* and *PTCH1* in HH39 control$^{+/+}$ and *ta*^{2} hindguts. (N) Counts for *PTCH1* puncta in HH39 control$^{+/+}$ and *ta*^{2} hindguts e: epithelium, sm: submucosal mesenchyme. Scale bars: (A-F, H-M) 100μm. Statistical analysis was performed utilizing Student's *t*-test (* denotes $P < 0.05$).
